# Oncogenic Ras deregulates cell-substrate interactions during mitotic rounding and respreading to alter cell division orientation

**DOI:** 10.1016/j.cub.2023.05.061

**Published:** 2023-06-20

**Authors:** Sushila Ganguli, Tom Wyatt, Agata Nyga, Rachel H. Lawson, Tim Meyer, Buzz Baum, Helen K. Matthews

**Affiliations:** 1 Laboratory for Molecular Cell Biology, University College London, Gower Street, London WC1E 6BT, UK; 2 Laboratoirè Matiere et Systemes Complexes, Université Paris Diderot, 10 rue Alice Domon et Léonie Duquet, Batiment Condorcet, 75013 Paris, France; 3 MRC Laboratory of Molecular Biology, Francis Crick Avenue, Cambridge CB2 0QH, UK; 4 School of Biosciences, University of Sheffield, Western Bank, Sheffield S10 2TN, UK; 5 UCL Cancer Institute, University College London, 72 Huntley Street, London WC1E 6DD, UK

## Abstract

Oncogenic Ras has been shown to change the way cancer cells divide by increasing the forces generated during mitotic rounding. In this way, Ras^V12^ enables cancer cells to divide across a wider range of mechanical environments than normal cells. Here, we identify a further role for oncogenic Ras-ERK signaling in division by showing that Ras^V12^ expression alters the shape, division orientation, and respreading dynamics of cells as they exit mitosis. Many of these effects appear to result from the impact of Ras^V12^ signaling on actomyosin contractility, because Ras^V12^ induces the severing of retraction fibers that normally guide spindle positioning and provide a memory of the interphase cell shape. In support of this idea, the Ras^V12^ phenotype is reversed by inhibition of actomyosin contractility and can be mimicked by the loss of cell-substrate adhesion during mitosis. Finally, we show that Ras^V12^ activation also perturbs division orientation in cells cultured in 2D epithelial monolayers and 3D spheroids. Thus, the induction of oncogenic Ras-ERK signaling leads to rapid changes in division orientation that, along with the effects of Ras^V12^ on cell growth and cell-cycle progression, are likely to disrupt epithelial tissue organization and contribute to cancer dissemination.

## Graphical abstract

**Graphic abstract:**
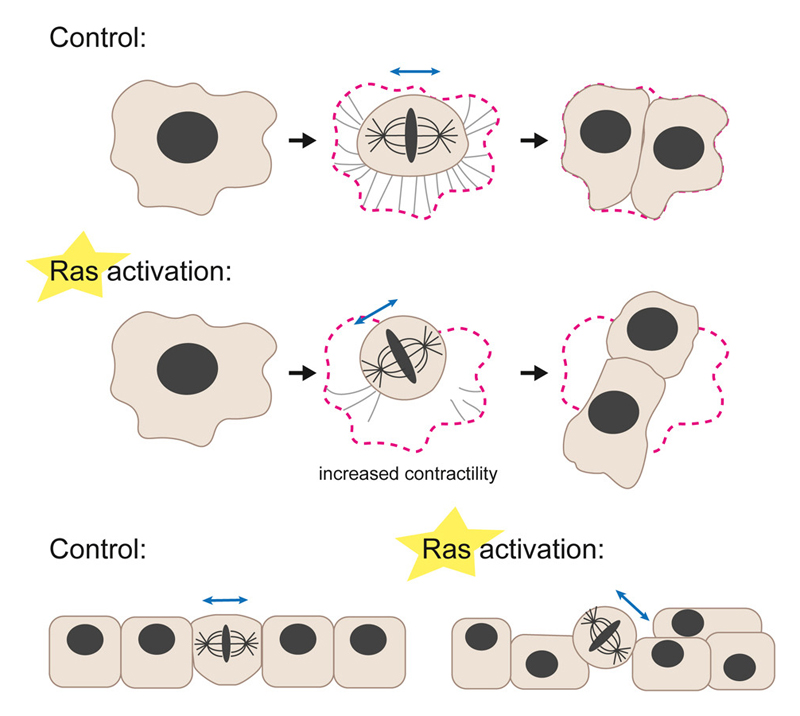

## Introduction

The Ras family of genes were among the first identified human oncogenes^1^ and are the most frequently mutated in cancer.^2^ Activation of Ras oncogenes results in the hyperactivation of proliferative signaling pathways, including the ERK and PI3K pathways, to promote growth, survival, and cell-cycle entry.^3,4^ Ras-ERK signaling also affects the dynamic organization of the actin cytoskeleton,^5,6^ leading to changes in cell morphology and behavior that aid invasion and metastasis.^7–10^ In recent work, we showed that oncogenic Ras-ERK signaling also impacts the shape and mechanics of cells entering mitosis.^11^ Entry into mitosis is accompanied by large-scale changes in cell shape and mechanics, both in cell culture and in a tissue context. These begin with mitotic cell rounding, a process driven by reorganization of the actin cytoskeleton, decreased substrate adhesion, and osmotic swelling.^12^ Mitotic cell rounding has been shown to be a functionally important part of the division process because it creates space for the mitotic spindle, for the alignment of chromosomes at metaphase, and for well-regulated chromosome segregation.^13,14^ We previously showed that the activation of oncogenic Ras-ERK signaling increased cell rounding in early mitosis. These changes in cell geometry were accompanied by alterations in cell mechanics that limit the DNA segregation errors observed in confined cell division.^11^

Mitotic rounding is dependent on the partial de-adhesion of cells from the substrate.^15–18^ As cells round, they remain attached to the substrate via an array of thin, actin-rich retraction fibers.^19^ The forces communicated by these retraction fibers have been proposed to position and orient the mitotic spindle,^20–22^ which determines the axis of cell division. In addition, mitotic integrin-mediated adhesion plays an important role in orienting the spindle in the plane of the substrate^20–22^ to ensure that both daughter cells remain attached to the substrate after division.^22,23^ Retraction fibers also provide mitotic cells with a memory of their former interphase cell shape as daughter cells respread along retraction fibers^16^ to re-occupy the interphase footprint of their mother.^16,24^ Although the cues orienting division in epithelia are more complex, similar processes are likely to be important in this context because the regulation of division orientation and post-mitotic cell placement are both crucial for the maintenance of normal tissue architecture.^25^

Having previously shown that activation of Ras oncogenes can alter the dynamics of mitotic rounding, in this paper we set out to investigate how oncogenic Ras influences mitotic exit. Our analysis reveals that the expression of oncogenic HRas^G12V^ rapidly alters many aspects of mitotic exit, including division orientation and respreading dynamics. These effects likely result from the impact of Ras^V12^ on actomyosin contractility during mitotic rounding because Ras^V12^ induces severing of the retraction fibers that normally guide spindle positioning and which provide a memory of the interphase cell shape. In support of this idea, the Ras^V12^ phenotype is reversed by inhibition of actomyosin contractility and can be mimicked by the loss of cell-substrate adhesion during mitosis. Oncogenic Ras also impacts the division orientation of epithelial cells cultured within 2D monolayers and 3D spheroids, implying further roles for Ras^V12^ in disrupting cell division. Together, these findings show how activation of a single oncogene can directly alter the spatial control of cell division as a relatively early event in oncogenesis.

## Results

### Ras-ERK signaling alters the dynamics of post-mitotic respreading

**Figure 1:**
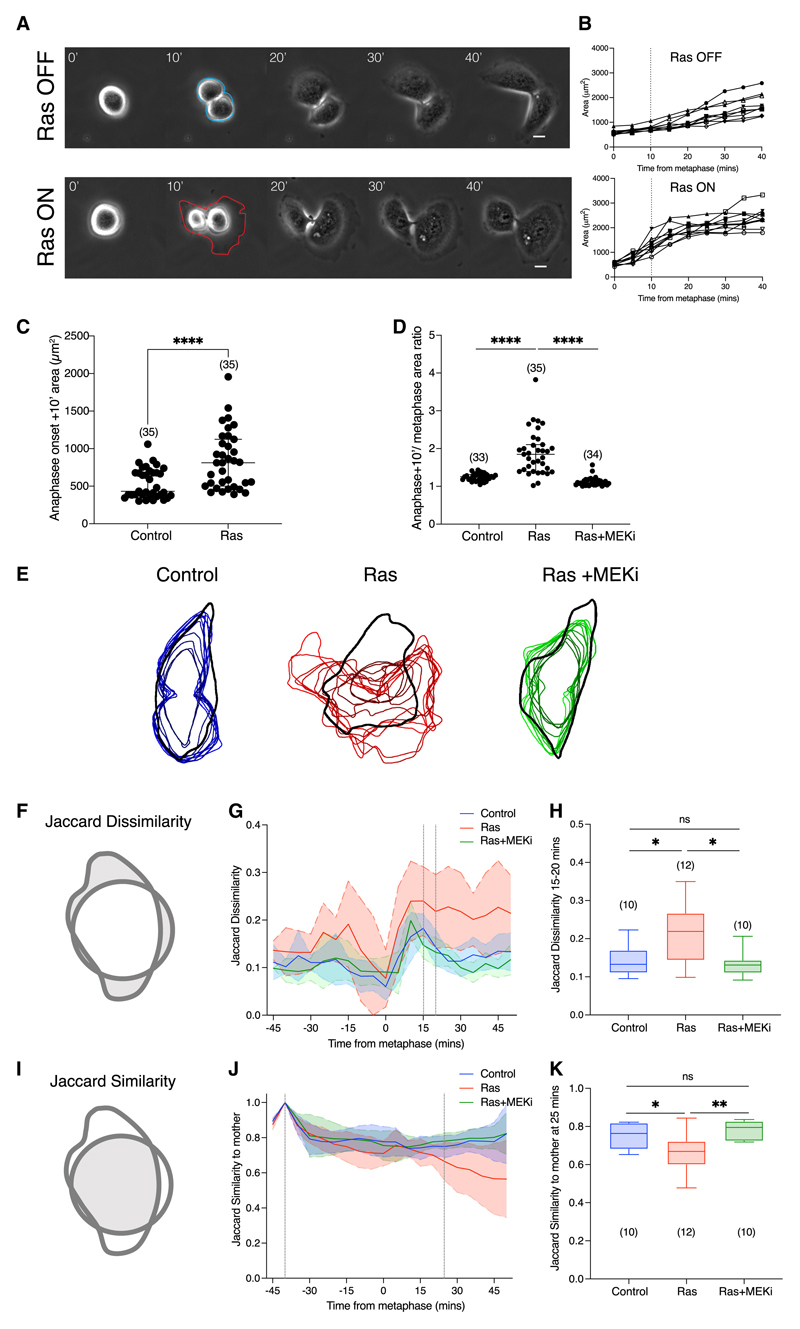
Ras-ERK signaling alters the dynamics of post-mitotic respreading (A) Representative phase contrast images of ER-Ras^V12^ cells exiting division following ethanol or 4-OHT treatment. Time in minutes is aligned so that t = 0 is the frame 5 min before the first evidence of anaphase elongation or visible chromosome separation. Metaphase and combined daughter cell areas were manually segmented from images as illustrated; ethanol (blue) and 4-OHT (red). Scale bars represent 10 μm. (B) Quantification of cell area for 10 ER-Ras^V12^ cells exiting mitosis following ethanol or 4-OHT treatment, as described in (A). Measurements were taken from phase-contrast time-lapse microscopy images of cells every 5 min following 5–15 h of treatment. Dotted lines indicate 10 min after the onset of anaphase, from which statistical analysis is calculated in (C). (C) Plot of cell area 10 min following the onset of anaphase of ER-Ras^V12^ cells treated with ethanol or 4-OHT. P values calculated using Mann-Whitney test. N = 3 experiments. (D) Plot of the ratio of cell area (10 min following anaphase onset/metaphase) for ER-Ras^V12^ cells following ethanol, 4-OHT, or 4-OHT + 10 mM selumetinib (MEKi) treatment. P values calculated using Mann-Whitney test. N = 3 experiments. (E) Cell outlines as cells respread post-mitosis with respect to the mother cell shape. MCF10A-LifeAct-GFP-ER-Ras^V12^ cells were treated with ethanol, 4-OHT, or 4-OHT plus 10 mM selumetinib for 5–15 h prior to time-lapse bright-field and fluorescence imaging at 5-min intervals. Representative dividing cells were manually segmented at interphase (black), defined using the bright-field channel at 15 min before nuclear envelope breakdown (NEB). Post-mitotic respreading cell outlines were segmented at 5-min intervals for 10 consecutive frames from the final frame of metaphase following ethanol (blue), 4-OHT (red), or 4-OHT plus MEKi (green). Image overlays were produced using CellProfiler software as described in STAR Methods. (F) Diagram showing the definition of the Jaccard dissimilarity measurement. Gray indicates the non-overlapping area of two cell shapes. (G) Graph to show the Jaccard dissimilarity measurement with contributions of area, centroid displacement, and orientation change removed as cells progress through mitosis, comparing cell shapes between consecutive time points (5-min intervals). Dotted lines indicate 15 and 20 min following anaphase onset, used for statistical analysis in (H). n = 10 cells in each condition. (H) Box-whisker plot to show the Jaccard dissimilarity between 15- and 20-min time points following anaphase onset for all three conditions. P values calculated using Mann-Whitney test. (I) Diagram to show the definition of the Jaccard similarity measurement. Gray indicates the overlapping area of two cell shapes. (J) Graph to show the Jaccard similarity–with contributions of area, centroid displacement, and orientation change removed–of cells as they progress through mitosis at consecutive time points compared with the mother cell. Dotted lines indicate 15 min before NEB and 25 min following the onset of anaphase, used for statistical analysis in (K). n = 10 cells in each condition. (K) Box-whisker plot to show the Jaccard similarity comparing time points 25 min following anaphase onset with the mother cell for all three conditions. P values calculated using Mann-Whitney test. See also Figure S1 and Videos S1 and S2.

To investigate the impact of short-term oncogenic Ras signaling on dividing cells, we used the previously validated, tamoxifen-inducible estrogen receptor (ER) HRas^V12^ fusion system^11,26^ to transiently activate HRas^V12^ in the human, non-transformed epithelial cell line, MCF10A.^27^ In this system, the addition of 4-OH-tamoxifen (4-OHT) leads to the rapid activation of ERK and PI3K signaling,^11,26^ altering mitotic cell rounding within 5 h of Ras^V12^ expression.^11^ To determine how this impacts mitotic exit, we used bright-field time-lapse microscopy to follow unlabeled, asynchronous cells as they progressed beyond metaphase to divide and respread (Figure 1A). We then measured metaphase cell area and combined daughter cell areas to assess respreading dynamics in the presence or absence of Ras^V12^ induction (Figure 1B). This analysis revealed that Ras^V12^ activation accelerates the rate of post-mitotic respreading (Figure 1B) and results in a significant increase in respread cell area within 10 min of the onset of anaphase (Figure 1C).

As the effects of Ras^V12^ on mitotic rounding are mediated through ERK signaling, we next tested whether the change in rate of post-mitotic respreading was sensitive to MEK inhibition. For this analysis, inducible ER-Ras^V12^ cells were treated with ethanol (control), 4-OHT, or 4-OHT, plus the previously validated MEK inhibitor, selumetinib.^11^ This treatment interferes with mitotic rounding.^11^ To control for cell shape as a potential confounding factor, we normalized the analysis by calculating the ratio of cell area at metaphase relative to cell area 10 min after anaphase onset (post-anaphase cell area / metaphase cell area). This analysis revealed a significant increase in the rate of respreading in early anaphase following Ras^V12^ expression (independent of its impact on mitotic rounding), which was reversed with inhibition of ERK signaling (Figure 1D). Thus, Ras-ERK signaling increases the rate at which mitotic cells change shape by accelerating the rate of both mitotic rounding and post-mitotic cell respreading.

Ras^V12^ expression also visibly altered the shape of cells as they respread. To examine this phenotype in more detail, we used LifeAct-GFP labeled cells to enhance cell segmentation and tracking of the cell margin as cells exited mitosis (Figure S1A; Videos S1 and S2). In this experiment, ER-Ras^V12^-LifeAct-GFP cells were treated with ethanol, 4-OHT, or 4-OHT plus selumetinib, immediately prior to live bright-field and fluorescence imaging, and cell shapes were segmented in dividing cells (Figures 1E and S1B). While control cells and cells treated with 4-OHT + MEK inhibitor were found to maintain a consistent shape as they exited mitosis, cells expressing oncogenic Ras^V12^ during post-mitotic respreading had a variable shape–indicating that Ras-ERK signaling has a dramatic impact on the dynamics of cell shape changes that accompany mitotic exit.

As a simple quantitative measure of shape similarity to analyze respreading dynamics, we used the Jaccard index,^28^ which measures the relative overlap of different shapes (Figure This was then extended to define a Jaccard dissimilarity index as the area of the non-overlapping regions of the two shapes (Figure 1F). Changes in Jaccard dissimilarity can arise from changes in cell area, displacement, or orientation, as well as cell shape. We found that cell area change, displacement, and orientation are all increased by oncogenic Ras (Figures S1C–S1E). Therefore, in order to limit this measure to changes in cell shape, the contribution of area, displacement, and orientation were removed by scaling, translating, and rotating the images (see STAR Methods). In this way, the analysis revealed that Ras^V12^ activation significantly increases the rate at which cells change shape as they exit mitosis (Figures 1G and 1H).

In cell culture models of division, daughter cells tend to take up the shape of the mother cell footprint.^29^ Previous work has shown that this depends on the integrin-extracellular matrix (ECM) adhesions and retraction fibers that persist during mitosis.^19^ These act as a physical memory of interphase shape and guide daughter cell respreading.^16,17,24^ In line with these previous studies, control and MEK-inhibited cells adopted the same shape as the mother cell as they respread (Figure 1E). By contrast, cells expressing Ras^V12^ tended to escape the confines of the mother cell footprint. We quantified these effects using the Jaccard similarity index (Figure 1I) adapted to remove changes in area, displacement, and orientation. As control and MEK-inhibited cells started to exit mitosis, the Jaccard similarity increased, reflecting their ability to take up the mother cell shape (Figure 1J). However, over the same period, we also observed a significant decrease in the similarity of Ras^V12^ daughter cell footprint with the mother cell footprint (Figure 1K). This is consistent with previous findings, which showed that HRas induces high motility in normally non-motile HeLa cells, and thus reduces adherence to mother-cell shape following division.^24^ Similarly, in this system, Ras^V12^ expression increased interphase cell motility (Figure S1F). Taken together, these data show that short-term expression of oncogenic HRas increases the dynamics of cell spreading in cells leaving mitosis and decreases the likelihood of daughter cells taking up their mother cell’s footprint.

### Ras-ERK signaling induces the asymmetric respreading of daughter cells

**Figure 2:**
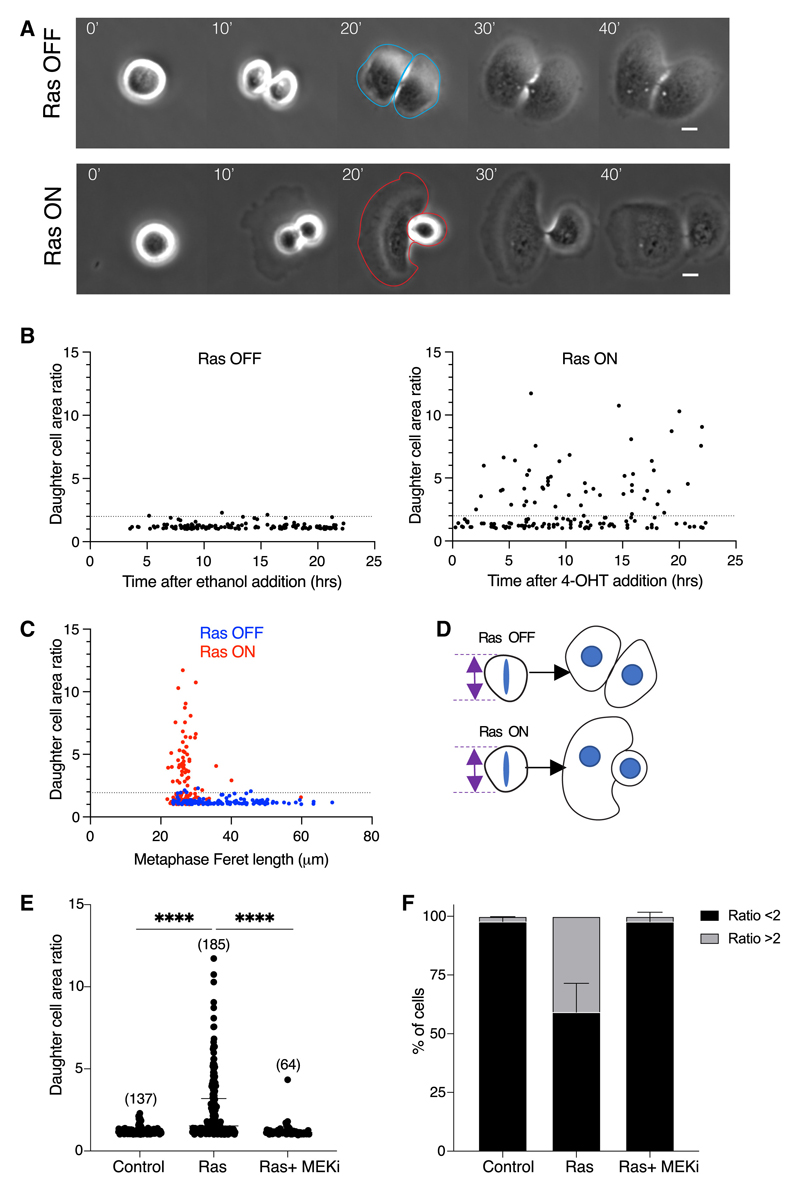
Ras-ERK signaling induces the asymmetric respreading of daughter cells (A) Representative phase contrast images of ER-Ras^V12^ cells exiting division following ethanol or 4-OHT treatment. Cells were imaged following 5–15 h of treatment at 5-min intervals. Time in minutes is aligned so that t = 0 is the frame 5 min before evidence of anaphase elongation or visible chromosome separation. Individual daughter cell areas are segmented from images as illustrated; ethanol (blue) and 4-OHT (red). Scale bars represent 10 μm. (B) Plot of the daughter cell area ratio of individual ER-Ras^V12^ cells dividing against time after ethanol addition (left-hand graph) or 4-OHT addition (right-hand graph). Measurements of individual daughter cell areas were taken at 20 min following the onsetof anaphase. The ratio was measured as larger/smaller. Dotted line indicates daughter cell area ratio of 2. N = 2 experiments. (C) Plot of the metaphase cell length (Feret) against daughter cell area ratio of individual ER-Ras^V12^ cells dividing following ethanol (blue) or 4-OHT (red) treatments. Dotted line indicates daughter cell area ratio of 2. N = 2 experiments. (D) Diagram depicting how control cells that are able to round up at metaphase to the same extent as Ras^V12^-activated cells continue to divide symmetrically. (E) Plot ofdaughter cell area ratio ofER-Ras^V12^ cells following ethanol, 4-OHT, or 4-OHT plus selumetinib treatment. Measurements were taken from phase contrast imaging of ER-Ras^V12^ cells as described in (A). P values calculated using Mann-Whitney test. N = 3 experiments. (F) Graph showing the percentage of cells from the data in (E) that divide with a daughter cell area ratio of < or ≥ 2. Error bars show SD. N = 3 experiments. See also Figure S2.

Through studying the dynamics of cell respreading during mitotic exit, we also observed that Ras^V12^ expression frequently causes differences in the rate at which the two daughter cells generated at division respread. To quantify this asymmetry in the respreading process, bright-field imaging was used to follow divisions in unlabeled control and Ras^V12^-expressing cells (Figure 2A). Daughter cell areas were manually segmented at 20 min following anaphase onset–a time when respreading has mostly been completed (Figures 1B and S1C). The ratio of the two daughter cell areas (larger/smaller) was then calculated and plotted against time (Figure 2B). While ethanol-treated control cells tended to respread symmetrically over 23 h of imaging, maintaining a ratio of <2 in 97.5% of divisions (Figure 2B, left-hand graph), 37% of cells expressing Ras^V12^ underwent asymmetric respreading (ratio ≥2) (Figure 2B, right-hand graph). Importantly, these asymmetries resolved in the following inter-phase (Figure S2B), ruling out this being due to an asymmetric partitioning of cell volume or content. Remarkably, the asymmetry in the ability of daughter cells to respread was observed as early as 3 h after 4-OHT addition (Figure 2B), yet persisted following prolonged HRas expression, as an MCF10A cell line constitutively expressing HRas^V12^ over many generations also exhibited respreading asymmetry, a phenotype that was reversed upon MEK inhibition (Figure S2A).

Having previously demonstrated an impact of oncogenic Ras-ERK signaling on mitotic rounding,^11^ it was important to test whether the observed defects in division symmetry reflected differences in the extent of mitotic rounding induced by Ras^V12^ expression. To do so, we plotted metaphase cell length against daughter cell area ratio for control and Ras^V12^-expressing cells (Figure 2C). This proved not to be the case. Thus, while asymmetric divisions were frequently seen in Ras^V12^-expressing cells, they were rarely observed in divisions of near-spherical control cells (Figures 2C and 2D). Importantly, the effect of Ras^V12^ on division symmetry was abolished by treatment with the MEK inhibitor (Figures 2E and 2F).

### Activation of oncogenic Ras causes misalignment of the mitotic spindle

**Figure 3:**
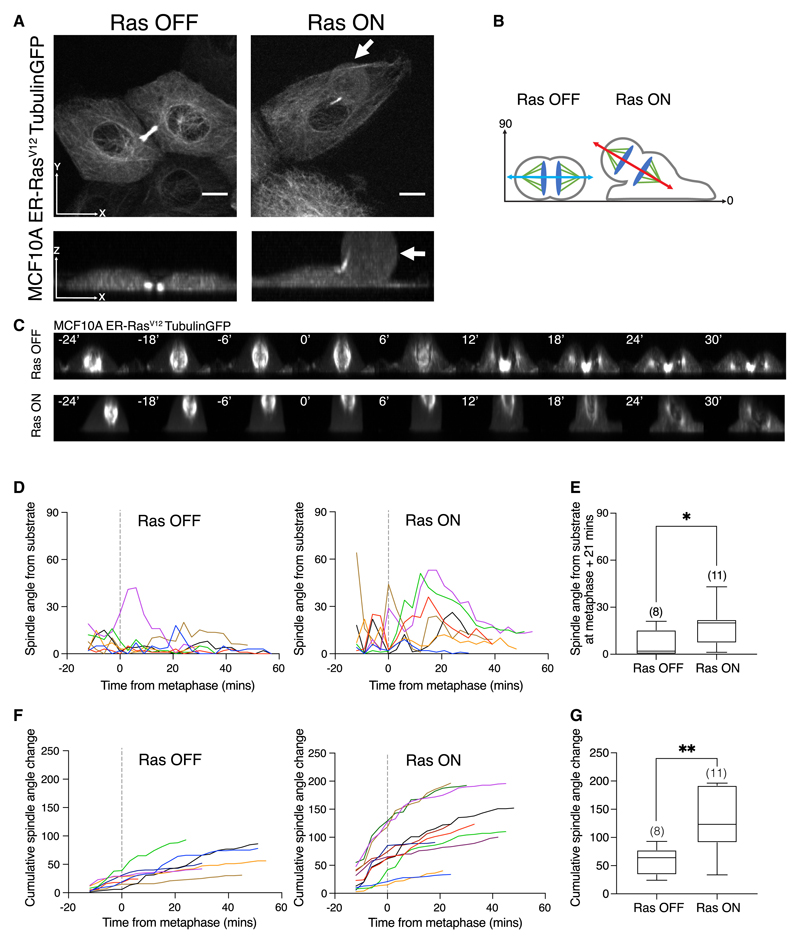
Activation of oncogenic Ras causes misalignment of the mitotic spindle (A) Representative images of MCF10A ER-Ras^V12^ cells labeled with tubulin-GFP following a 5-h treatment with ethanol or 4-OHT. Images are displayed as maximum projections in x-y and x-z. Arrows indicate an apically positioned daughter cell. Scale bars represent 10 μm. (B) Schematic to show spindle alignment relative to the substrate according to Ras^V12^ activation. (C) Representative time-lapse images of MCF10A ER-Ras^V12^ cells labeled with tubulin-GFP progressing through mitosis following a 5-h treatment of ethanol or 4-OHT, taken at 3-min intervals. Images are presented as maximum projections in x-z. Time is aligned so t = 0 represents the frame 3 min before anaphase spindle elongation. Manual segmentation of the axis between the two spindle poles from the time of the first appearance of the mitotic spindle enabled calculation of the angle of the spindle relative to the substrate (x axis = 0°). (D) Quantification of the spindle angle relative to the substrate as described in (C) over time for 7 individual MCF10A ER-Ras^V12^-tubulin-GFP cells following a 5-h treatment of ethanol or 4-OHT. Dotted lines at t = 0 represent the frame 3 min before anaphase spindle elongation. (E) Box-whiskerplottoshowthespindleangleat21 min followingtheonsetofanaphaseforER-Ras^V12^-tubulin-GFPcellsfollowinga5-htreatmentofethanolor4-OHT. P values calculated using Mann-Whitneytest. (F) Graphs to show the cumulative spindle angle from substrate change as cells progress through mitosis at 3-min intervals for individual cells, as described in (C). (G) Box-whisker plot to show the cumulative spindle angle change for ER-Ras^V12^-tubulin-GFP cells following a 5-h treatment of ethanol or 4-OHT. P values calculated using unpaired student’s t test.

Asymmetric respreading could arise as a consequence of alterations in mitotic spindle orientation, as the plane of cell division in animal cells is determined by the orientation of the spindle.^23^ To examine this further, we constructed a MCF10A ER-HRas^V12^ cell line that stably expresses tubulin-GFP to visualize mitotic spindle dynamics during mitosis. Confocal live cell imaging was then used to follow tubulin-GFP in control and Ras^V12^-expressing cells in 3D as they divided and respread (Figure 3A). As observed in X-Z cross-sections, the spindle and mid-body of Ras^V12^-activated cells were frequently aligned at an oblique angle relative to the substrate. As a result, upon exit from mitosis, only one of the two Ras^V12^-expressing daughter cells retained contact with the substrate, enabling it to respread rapidly (Figures 3A and 3B).

To further characterize the impact of Ras^V12^ activation on the positioning of the mitotic spindle relative to the plane of the substrate, live cell imaging was again used to follow tubulin-GFP-labeled spindles in ER-Ras^V12^ cells treated with ethanol or 4-OHT as they progressed through mitosis (Figure 3C). By imaging cells in the X-Z plane, we were able to show that spindles in Ras^V12^-expressing cells have an unstable position and are frequently tilted at an angle of >30° from the substrate (Figure 3D). While some control cells transiently exhibited tilted spindles (>30°), such errors tended to be corrected soon after mitotic exit, resulting in interphase daughter cells positioned side by side on the substrate within 21 min of anaphase (Figure 3E).

By contrast, cells expressing Ras^V12^ exhibited large defects in spindle orientation early in mitosis (>30°) and took much longer to correct these defects following mitotic exit (Figures 3D and 3E). The combined effects of these processes led to a significant increase in the cumulative movement of spindles in Ras^V12^-activated cells relative to the control (Figures 3F and 3G).

### Ras activation induces breakages in retraction fibers

**Figure 4:**
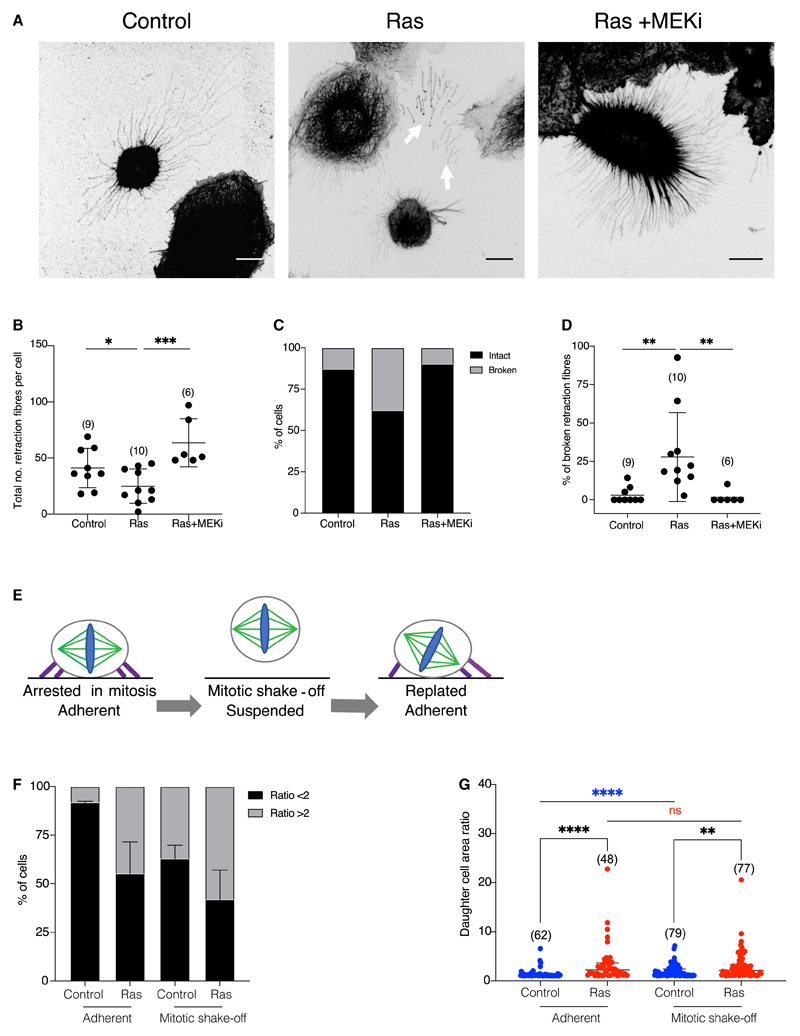
Ras activation induces breakages in retraction fibers (A) Immunofluorescence images of MCF10A-ER-RasV12 cells in metaphase following a 5-h treatment of ethanol, 4-OHT, or 4-OHT + 10 mM selumetinib (MEK inhibitor). Cells are stained for monomeric α-tubulin. Images are displayed as maximum projections. Arrows indicate breakages in retraction fibers. Scale bars represent 10 μm. (B) Quantification of the total number of intact retraction fibers in single cells from the conditions described in (A). P values calculated using unpaired student’s t test. (C) Graph showing the percentage of cells as described in (A), with evidence of any broken retraction fibers. n = control (15), Ras (19), Ras + MEKi (10). (D) Quantification of the percentage of broken retraction fibers in single cells from the conditions described in (A). P values calculated using Mann-Whitney test. (E) Schematic to illustrate the mitotic shake-off protocol. ER-RasV12 cells were treated with 10 mmol S-trityl-L-cysteine (STLC) and ethanol or tamoxifen for 15 h prior to “mitotic shake-off.” Mitotic cells are then dislodged by mechanical force into suspension. Upon removal of STLC, cells are then replated onto fibronectin-coated glass dishes in ethanol or tamoxifen-containing media and immediately imaged using bright-field time-lapse microscopy at 5-min intervals as they exit mitosis. (F) Graph showing the percentage of cells that divide with a daughter cell area ratio of < or ≥ 2 according to whether they are subjected to mitotic shake-off or not. Adherent cells are plated on fibronectin-coated glass dishes for 24 h and treated with ethanol or 4-OHT immediately prior to imaging. Mitotic shake-off cells were treated as described in (E). Measurements of daughter cell areas at 20 min following the onset of anaphase were taken for individual cells and the percentage of cells with a daughter cell area ratio of < or ≥ 2 was calculated. Error bars show SD. N = 3 experiments. (G) Individual daughter cell area ratio measurements from the experiment described in (F). P values calculated using Mann-Whitney test. See also Figures S3 and S4.

Although adhesions are remodeled as cells pass into and out of mitosis,^30^ mitotic cells maintain physical connections with the extra-cellular environment through retraction fibers.^18,19^ These structures, formed during mitotic rounding, are tethered to the substrate at their tips by modified integrin-based structures that lack many of the components associated with interphase adhesions.^16,17^ Importantly, these mitotic adhesive structures have been implicated in positioning of the mitotic spindle^20,21^ and have been shown to guide post-mitotic respreading.^16,17^ When we imaged retraction fibers in fixed metaphase cells (Figures 4A and S3), we found a small but significant reduction in the numbers of retraction fibers per cell in Ras^V12^-expressing cells compared with those in the control. This effect was reversed following treatment with a MEK inhibitor (Figure 4B). In addition, we frequently observed severed retraction fibers in Ras^V12^-activated cells (Figure 4A). The proportion of cells with severed retraction fibers was significantly higher in Ras^V12^-activated cells compared with controls cells or cells treated with a MEK inhibitor (Figures 4C and 4D). This suggests the possibility that the effects of Ras^V12^ on mitotic exit are mediated by the breakage of retraction fibers severing the cell’s connection with the substrate. To test whether the loss of retraction fibers could be responsible for the defects observed at mitotic exit, we dislodged cells from the substrate directly using mechanical force. These suspension cells were then replated and imaged as they exited mitosis, divided, and re-spread on a new substrate in the absence of retraction fibers (Figures 4E and S4A). Again, the ratio of the spread area of the two daughter cells was measured to assess division symmetry 20 min after the onset of anaphase. Control cells replated from suspension displayed a marked increase in division asymmetry relative to their undisturbed counterparts (Figure 4F), as expected if these cells are unable to accurately determine the plane of the substrate. However, mitotic spindle orientation in these cells was gradually corrected, as cells exited mitosis and re-spread (Figure 4G). By contrast, levels of spindle misorientation and division asymmetry remained high in Ras^V12^-expressing cells, irrespective of whether or not they had been subjected to mitotic shake off (Figures 4F and 4G). Furthermore, unlike control cells, cells expressing oncogenic Ras with misaligned spindles were unable to correct this defect as they respread (Figure 4G). Similar behavior was observed for cells plated on hydrogels of different stiffnesses (Figure S4B). On gels with a stiffness of between 2 and 12 kPa, upon which cells were able to spread, nearly all control cells were seen dividing parallel to the substrate, while around 30% of Ras^V12^-activated cells divided perpendicular to the substrate. By contrast, on very soft gels (0.7 kPa), which prevent both control and Ras^V12^-expressing cells from spreading and from forming retraction fibers, divisions were frequently misoriented in the x-z axis. Taken together, these data demonstrate that, while the maintenance of mitotic cell-substrate interactions is crucial for correct spindle orientation and symmetrical respreading in normal cells grown on a substrate, the ability of cells to read the substrate is compromised by the expression of oncogenic Ras.

### The effects of oncogenic Ras on mitotic exit depend on actomyosin contractility

**Figure 5:**
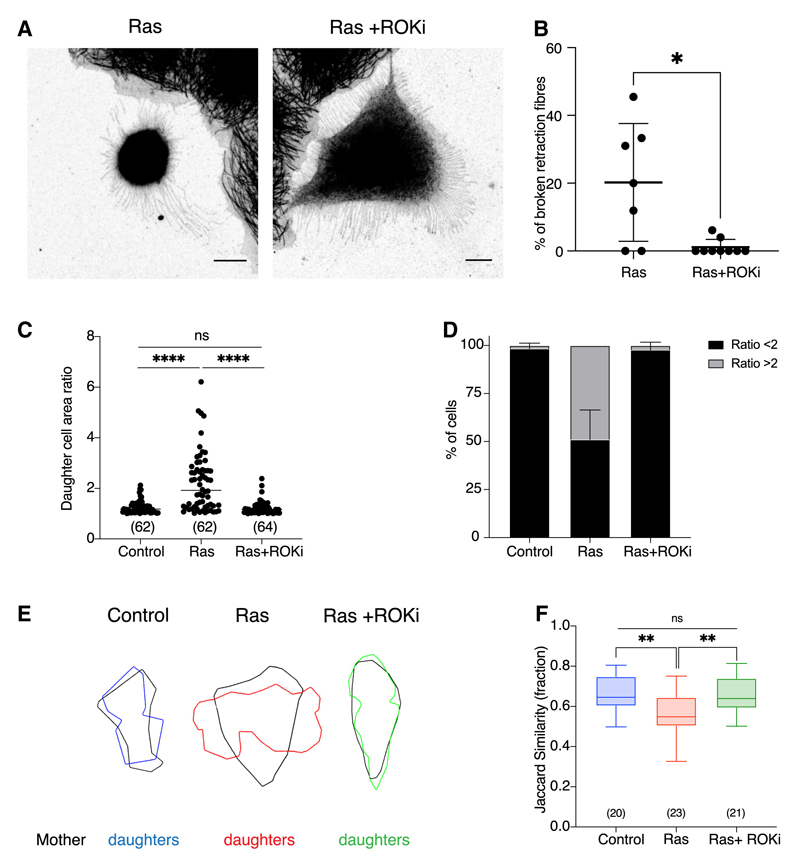
The effects of oncogenic Ras on mitotic exit depend on actomyosin contractility (A) Immunofluorescence images of MCF10A-ER-Ras^V12^ cellsin metaphase following a 5-h treatmentof 4-OHT or 4-OHT + 25 μm Y27632 (ROCK inhibitor). Cells are stained for monomeric μ-tubulin. Images are displayed as maximum projections. Scale bars represent 10 μm. (B) Quantification of the percentage of broken retraction fibers in single cells from the conditions described in (A). n = Ras (7), Ras + ROKi (9). P values calculated using Mann-Whitney test. (C) Quantification of daughter cell area ratio for ER-Ras^V12^ cells following ethanol, 4-OHT, or 4-OHT plus ROCK inhibitor treatment. Measurements taken from bright-field time-lapse microscopy images as previously described. P values calculated using Mann-Whitney test. N = 3 experiments. (D) Graph showing the percentage of cells from the data in (C) that divide with a daughter cell area ratio of < or #x2265; 2. Error bars show SD. N = 3 experiments. (E) Overlay of mother and daughter cell outlines. Representative cells from the experiment described in (C) were manually segmented at NEB minus 15 min (mother) and 15 min following the onset of anaphase (daughters). (F) The Jaccard similarity index (shape contribution only) quantifying the overlap between mother and daughter cell shapes as defined in (E). P values calculated using unpaired student’s t test. See also Figure S5.

Because oncogenic Ras increases actomyosin contractility to drive enhanced mitotic rounding,^11^ it seemed possible that the increased actomyosin contractility in Ras^V12^-expressing cells entering mitosis might cause retraction fiber severing. To test this idea, we imaged cells dividing in the presence or absence of an inhibitor of Rho kinase (ROCK) to reduce cortical contractility.^31,32^ As expected, ROCK inhibition increased cell area in both control and Ras^V12^-expressing cells in meta-phase^31,32^ and during respreading (Figures S5A–S5C). The ROCK inhibitor also prevented the severing of retraction fibers (Figures 5A, 5B, and S5D), increased the total number of retraction fibers tethering mitotic cells to the substrate (Figure S5E), and restored normal division orientation and post-mitotic re-spreading in Ras^V12^-activated cells (Figures 5C and 5D). Finally, the addition of ROCK inhibitor restored the ability of daughter cells to spread into their mother’s cell footprint, as measured by the Jaccard similarity index (Figures 5E and 5F). These data show that actomyosin contractility is crucial for the effects of Ras^V12^ activation on cell division orientation and post-mitotic respreading.

### Oncogenic Ras de-regulates division orientation in cell monolayers and spheroids

**Figure 6:**
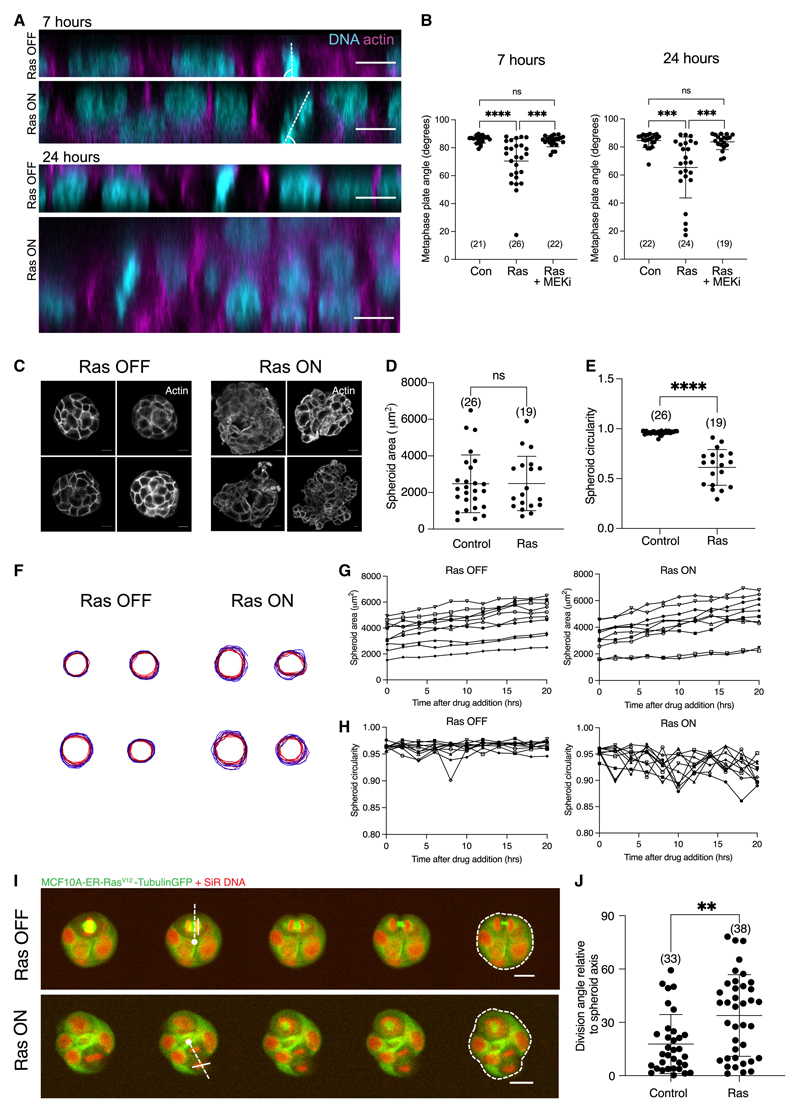
Oncogenic Ras deregulates division orientation in cell monolayers and spheroids (A) Orthogonal sections of immunofluorescence images of MCF10A-ER-HRas^V12^ cells plated as a confluent monolayer on a 12 kPa collagen-coated poly-acrylamide hydrogel following 7 or 24 h of treatment with ethanol or 4-OHT treatment. Dotted lines indicate metaphase plate used to measure angle in (B). (B) Quantification of the angle between the line of the metaphase plate and the substrate in cells treated with 7 or 24 h of ethanol, 4-OHT, or 4-OHT and selumetinib (MEKi). P values calculated using unpaired student’s t test. N = 3 experiments. (C) Immunofluorescence images of MCF10A-ER-HRas^V12^ spheroids. Spheroids were cultured in Matrigel for 24 h following plating of a single-cell suspension. Spheroids were then fixed following 48 h of ethanol or 4-OHT treatment. Spheroids have been stained with Phalloidin-TRITC. Images are displayed as a single confocal plane. Scale bars represent 10 μm. (D) Graph to show the spheroid cross-sectional area (μm^2^) of fixed MCF10A-ER-HRas^V12^ spheroids following 48 h of ethanol or 4-OHT treatment. P values calculated using Mann-Whitney test. N = 2 experiments. (E) Graph to show the spheroid circularity of fixed MCF10A-ER-HRas^V12^ spheroids following 48 h of ethanol or 4-OHT treatment. P values calculated using MannWhitney test. N = 2 experiments. (F) Spheroid outlines during 0–20 h treatment with ethanol or 4-OHT. MCF10A-ER-Ras^V12^-Tubulin-GFP spheroid culture was carried out as described in STAR Methods. MCF10A-ER-Ras^V12^-Tubulin-GFP spheroids were imaged following treatment with ethanol or 4-OHT using time-lapse fluorescence imaging every 5 min. Spheroid outlines were manually segmented at 2-h intervals following ethanol or 4-OHT addition. (G) Quantification of spheroid area for 10 individual MCF10A-ER-Ras^V12^-Tubulin-GFP spheroids following ethanol or 4-OHT treatment, as described in (F). Spheroids were manually segmented every 2 h following ethanol or 4-OHT addition. (H) Quantification of spheroid circularity for 10 individual MCF10A-ER-Ras^V12^-Tubulin-GFP spheroids following ethanol or 4-OHT treatment, as described in (F). Spheroids were manually segmented every 2 h following ethanol or 4-OHT addition. (I) Representative time-lapse images of MCF10A ER-Ras^V12^-tubulin-GFP spheroids labeled with SiR-DNA following treatment with ethanol or 4-OHT taken at 5-min intervals. Images are presented as single confocal plane images. Dotted lines indicate the axis from the center of the spheroid to the periphery intersecting the center of the mitotic spindle. Solid white lines indicate the axis of the anaphase chromosomes. Dotted contour lines demonstrate manual segmentation of spheroid outlines as described in (F). Scale bars represent 20 μm (J) Quantification of cell division angle in MCF10A ER-Ras^V12^-Tubulin-GFP spheroids relative to the spheroid edge. The spheroid and division axes were manually segmented as illustrated in (I). A line from spheroid centroid to the periphery intersecting the dividing cell and a line parallel to the anaphase chromosomes were drawn. The Feret angle (0 = x axis) is calculated for each, and from these, quantification of the division axis relative to the spheroid axis is calculated. P values calculated using Mann-Whitney test. N = 2 experiments. See also Videos S3 and S4.

The work described so far focused on the effects of Ras in isolated cells, where contact with the substrate is a key factor in the regulation of spindle orientation.^20,21^ To investigate how oncogenic Ras impacts division orientation within the context of a collective, we imaged ER-HRas^V12^ cells dividing in a confluent MCF10A epithelial monolayer on top of collagen-coated hydrogels (Figure 6A), where cells assume a cuboidal shape. The activation of Ras^V12^ within such epithelial monolayers has previously been shown to induce a loss of tissue architecture.^33^ In agreement with this, we found that Ras^V12^ expression induced tissue multilayering after 24 h of 4-OHT treatment (Figure 6A). In this case, while control cells divided within the plane of the monolayer, with the angle between the metaphase plate and substrate close to 90°, Ras^V12^ expression led to a significant change in the spindle angle, causing many cells to undergo out-of-plane divisions (Figure 6B). Once again, the changes in division orientation induced by oncogenic Ras could be reversed by the addition of a MEK inhibitor (Figure 6B).

Finally, taking advantage of the ability to culture MCF10A cells in 3D,^34^ we examined the effect of Ras^V12^ activation on spheroids in Matrigel (Figures 6C-6E). In this system, 48 h of Ras^V12^ expression were sufficient to disrupt their normal spherical morphology (Figure 6C), resulting in cell clusters with a significantly decreased circularity (Figure 6E), without inducing changes in overall spheroid size (Figure 6D). To investigate how these morphological changes arise, we imaged these cell clusters live for 20 h following Ras^V12^ activation (Figures 6F-6J). These Ras^V12^-expressing spheroids became increasingly less circular over time (Figure 6H), without a change in overall growth rate (Figure 6G). To examine division orientation we imaged live MCF10A ER-HRas^V12^ cells expressing tubulin-GFP that had been labeled with SiR DNA (Figure 6I; Videos S3 and S4), and measured the angle between the anaphase chromosomes and the axis connecting the center of the spheroid to the margin as they divided (Figure 6J). While the orientation of control division angles was more variable in 3D spheroids than in 2D monolayers (Figures 6B and 6J), likely reflecting the variability of the adhesive and mechanical cues read by the spindle in 3D structures,^35^ the expression of Ras^V12^ was sufficient to induce a significant change in the angle of division–with many more Ras^V12^-expressing cells dividing perpendicular to the centroid-edge axis compared with controls (Figure 6J). Overall, these data reveal that oncogenic Ras induces changes to cell division orientation within cell collectives, as it does in single cells.

## Discussion

The precise regulation of cell division is crucial for the maintenance of normal tissue architecture.^23^ In this study, we show that the expression of the oncogene HRas^G12V^ leads to the misorientation of cell division and to defects in post-mitotic cell respreading. In earlier work, we showed that Ras^V12^ activation increases actomyosin contractility to accelerate mitotic cell rounding and to accentuate mitotic cell stiffness.^11^ The new data presented here show that this increased rounding force in early mitosis is also sufficient to sever the retraction fibers that anchor mitotic cells to the substrate. This loss of attachment to the substrate has two major effects. First, it leads to spindle misorientation relative to the substrate plane. Second, it prevents daughter cells from reoccupying the footprint of their mother following division. Remarkably, these effects of deregulated Ras signaling occur within a very short space of time following induced Ras^V12^ expression. They are also rapidly reversed by the addition of MEK inhibitors. Thus, while these changes could be due to Ras-ERK-mediated changes in transcription, their speed suggests the possibility of this being a more direct effect of signaling, as has been suggested by other studies implicating a direct role for ERK on actomyosin contrac-tility.^6–8,36^ The precise pathways by which Ras-ERK signaling alters actomyosin contractility in mitosis remain to be determined.

Integrin and actin-rich retraction fibers have been shown to maintain substrate attachment following mitotic rounding to guide spindle alignment.^20,21^ Precisely how cell-substrate adhesions regulate division orientation is not completely clear. The forces required for spindle orientation are thought to depend on interactions between astral microtubules and the cell cortex,^23^ which is patterned by polarity factors that integrate multiple cues from substrate adhesions, cell shape, and chromatin.^37,38^ This is also likely to apply to our system. Ras^V12^-activated cells form fewer retraction fibers and have more broken fibers than controls, something that is likely to have a profound impact on spindle orientation. At the same time, Ras^V12^ may also perturb the coupling between the retraction fibers that remain, cortical cues, and spindle microtubules in other ways. In addition, Ras^V12^ expression compromises spindle orientation even in cells that lack retraction fibers as a consequence of mitotic shake off (Figure 4F). It is notable that around 30%–40% of Ras^V12^-ex-pressing cells display division orientation defects, suggesting that spindle angle may not be completely randomized and that there may be some residual substrate sensing at play.

We find that Ras^V12^ activation also impairs the ability of daughter cells to take up the footprint of their mother after cell division, as has been previously observed in HeLa cells.^24^ This is another phenotype likely to result from changes to retraction fibers, which serve as a “memory” of mother cell shape in mitosis.^16,19^ Mali et al.^24^ have attributed such changes in re-spreading to the increased cell motility in oncogenic Ras-expressing cells. Because the rate of post-mitotic respreading and inter-phase motility were both increased in our study, Ras^V12^-induced changes in early interphase cell movement may also play a role here. In addition, decreased cortical tension in Ras-activated cells in interphase^11^ may also affect respreading rate.

Interestingly, the impact of oncogenic Ras on division orientation is independent of cell context. Thus, Ras^V12^ induces similar cell division orientation defects in single cells dividing on glass, where cells have extensive retraction fibers, in a confluent epithelial monolayer growing on an ECM-coated hydrogel, and in spheroids embedded within ECM. When epithelial cells are surrounded by neighbors, mitotic spindle orientation is influenced by multiple factors, including cell-cell and cell-ECM adhesions, polarity factors, and specialized structures such as tri-cellular junctions.^23–39,40^ All of these cues help cells in a monolayer to reliably divide in plane to maintain tissue architecture. In 3D spheroids, although similar cues are likely present, division orientation is more variable, likely reflecting measurement error together with variability in neighbors and local tissue architecture.^35^ The fact that Ras^V12^ perturbed normal cell division orientation in every case suggests that Ras^V12^ compromises the ability of cells to respond to normal spindle positioning cues in multiple ways. The idea that oncogenic Ras induces multiple downstream changes that influence spindle orientation is implied by the observation that Ras^V12^-expressing cells fail to correct errors in division orientation following mitotic shake off, while their control counterparts that also lack retraction fibers do. Furthermore, we were unable to observe retraction-fiber-like structures in cell monolayers or spheroids, implying that Ras is likely to have effects on division that extend beyond enhancing contractility to sever retraction fibers. It should be noted that it is not exactly clear how cell-ECM attachments are modulated during cell division in tissues in any model system. While cells in pseudo-stratified epithelia have been shown to maintain attachment to the basal lamina during mitotic rounding via thin basal processes that direct cleavage plane orientation,^41,42^ the extent to which these retraction fibers contribute to division orientation in tissues where additional cell-to-cell adhesions and polarity complexes are also at play remains to be determined.^40^

While the precise mechanisms by which Ras^V12^ impacts division in an epithelial monolayer or spheroid remains unclear, the Ras^V12^-induced changes to division orientation are likely to have important implications for tissue organization because oriented cell division is required to facilitate stress relaxation and to maintain cell packing^43,44^ and epithelial architecture.^23^ While errors in division orientation are tolerated in some tissues,^45^ departure from planar divisions can disrupt epithelial organization,^39,46^ leading to the idea that spindle misorientation might contribute to oncogenesis.^47^ Several studies support this hypothesis by showing how the disruption of planar spindle alignment can lead to cell delamination,^42^ the formation of tumor-like masses,^42,48^ as well as invasive cancer.^49^ In addition, there is an association between spindle misorientation phenotypes and oncogenic signaling.^48,50–52^ Oncogenic Ras has been shown to disrupt tissue structure, inducing 2D-3D transitions in tissue culture,^33^ and to induce the formation of tumorlike structures *in vivo*.^53,54^ We have observed similar disruption to tissue structure in MCF10A, with Ras^V12^ activation rapidly inducing bi-layering and a loss of spherical shape in epithelial cell clusters (Figure 6). These changes occur concurrently with alterations in division orientation but are likely to result from a combination of misoriented divisions and global changes in actomyosin contractility and cell-cell adhesion that occur downstream of Ras^V12^.^33^ It will be important to assess in detail to what extent altered cell division contributes to loss of tissue structure in these models and how these early consequences of oncogene signaling contribute to tumor formation and spread *in vivo*.

Our findings reveal a mechanism by which oncogenic mutations in Ras affect cell division orientation and post-mitotic shape changes, which have the potential to impact tissue organization during the earliest stages of tumor formation. This study focused on HRas^G12V^, a driver mutation in several cancers, including bladder and thyroid cancer and squamous cell carcinoma,^55^ where early HRas^G12V^ mutations drive abnormal tissue growth and folding.^56^ In our previous study, we found similar mitotic rounding phenotypes following oncogenic KRas and HRas expression.^11^ In future work, it will be important to test how well each model can be applied to cancers driven by other Ras isoforms. Nevertheless, taken together, our data show that Ras oncogenes act via ERK to accelerate actomyosin-dependent changes in cell shape in a way that impacts cell mechanics and the outcome of cell division. The consequences of these are likely be profound when combined with the influence of Ras on epithelial mesenchymal transition (EMT), cell growth and cell-cycle progression. How a single pathway is able to influence such a breadth of cell biology is unclear, but these multiple effects of Ras-ERK signaling on growth and division may explain why Ras oncogenes are such potent drivers of cancer development and progression.

## Star★Methods

**Table:** Key Resources Table

REAGENT or RESOURCE	SOURCE	IDENTIFIER
Antibodies		
Monoclonal anti-alpha-tubulin-FITC	Sigma	Cat# F2168 RRID: AB_476967
Chemicals, peptides, and recombinant proteins		
4-OH-tamoxifen	Sigma	Cat# H7904 CAS: 68392-35-8
Selumetinib	Selleckchem	Cat# S1008 CAS: 606143-52-6
Y-27632	Sigma	Cat# Y0503 CAS: 146986-50-7
S-Trityl-L-cysteine (STLC)	Sigma	Cat# 164739 CAS: 2799-07-7
SiR-DNA	Tebu-Bio	Cat# SC007
DAPI	Invitrogen	Cat# D3571
Phalloidin-TRITC	Sigma	Cat#P1951
Matrigel	Scientific Labwares	Cat# 354230
Fibronectin	Sigma	Cat# F1141
Collagen type I	Corning	Cat# 354249
Bind-Silane	Abbexa	Cat# abx082155
Sulfo-SANPAH	Sigma	Cat# 803332
Experimental models: Cell lines		
MCF10A-ER:hRas^G12V^	Laboratory of Julian Downward. Molina-Arcas et al.^26^	N/A
MCF10A-ER:hRas^G12V^+LifeAct-GFP	Matthews et al.^11^	N/A
MCF10A-ER:hRas^G12V^+Tubulin-GFP	This paper	N/A
Software and algorithms		
Jaccard index of intrinsic shape	This paper, Zenodo	https://doi.org/10.5281/zenodo.7948324
Fiji/ImageJ	Schneider et al.^57^	https://fiji.sc/
Prism 8	Graphpad	https://graphpad.com/
CellProfiler	Carpenter et al.^58^	https://cellprofiler.org
Arivis Vision4D	Zeiss	https://www.arivis.com/
Python	Python Software Foundation	https://www.python.org

## Resource Availability

### Lead contact

Further information and requests for resources and reagents should be directed to and will be fulfilled by the lead contact, Helen Matthews (h.k.matthews@sheffield.ac.uk).

### Materials availability

Cell lines generated in this study are available upon request from the lead contact.

### Data and code availability

- All data reported in this paper will be shared by the lead contact upon request
- All original code has been deposited at Zenodo and is publicly available as of the date of publication. DOIs are listed in the key resources table.
- Any additional information required to reanalyze the data reported in this paper is available from the lead contact upon request.

## Experimental Model And Subject Details

### Cell lines and culture

MCF10A-ER-HRas^V12^ (female, gift from J. Downward, Francis Crick Institute, London, UK)^26^ and MCF10A-ER-HRas^V12^ LifeAct-GFP^11^ (female) were cultured in phenol-free DMEM F-12 Glutamax with 5% charcoal-stripped horse serum (Invitrogen), 20ng/ml EGF (Peprotech), 0.5mg/ml Hydrocortisone (Sigma), 100ng/ml Cholera toxin (Sigma), 10μ>g/ml Insulin (Sigma), 1% Penstrep (Gibco) at 37°C with 5% CO_2_ Tubulin-GFP labelled lines were produced through infection with puromycin-resistant retrovirus. GFP positive cells were sorted using flow cytometry at the FACS facility at UCL Great Ormond Street Institute of Child Health to produce a polyclonal stable pool. All cell lines were authenticated using STR profiling (ATCC).

3D spheroid culture was established using Matrigel (Scientific Labwares).^34^ For ‘on-top’ spheroid culture, 45 μl Matrigel was distributed evenly on glass-bottomed dishes and polymerised at 37°C for 15 minutes. A single cell suspension was prepared in Assay Medium (DMEM F-12, 2% horse serum, 1% Pen/Strep, 0.5 μg/ml hydrocortisone, 100 ng/ml cholera toxin and 10 μg/ml insulin) containing 2% Matrigel and 5ng/ml EGF. 6000 cells were plated on top of solidified Matrigel in a volume of 400 μl. Spheroids were incubated for up to 4 days (see Figure Legends for details).

## Method Details

### Drug treatments

Ras^V12^ was activated in inducible lines by addition of 100nM 4-OH-tamoxifen (Sigma). The following small molecule inhibitors were used in this study: MEK inhibitor: 10μm Selumetinib (Selleckchem), ROCK inhibitor: 25μm Y27632 (Sigma). Where indicated, control treatments were performed with equivalent amounts of ethanol, DMSO or water. Details of treatment times are described in the figure legends.

To synchronise cells in metaphase for mitotic shake-off, cells were incubated with 10μm STLC (Sigma) for 15 hours. STLC was then washed out, using 3 washes, before replacing with fresh media as described in the figure legends.

### Live cell imaging

For live cell microscopy, cells were plated in fibronectin-coated, glass-bottomed plates (Mattek). Cells were plated 24 hours before imaging with the exception of the mitotic shake-off experiments where they were replated immediately before imaging. Wide field, brightfield time-lapse imaging at 37°C was carried out on a Nikon Ti inverted microscope at 5-minute intervals using a 20x (Plan Fluor ELWD Ph1 NA0.45, WD 7.4) or 40x (Plan Fluor ELWD Ph2 NA0.6, WD 3.7) objective. Live confocal imaging at 37°C was carried out on the 3i spinning disc confocal microscope using the 63x oil objective (Plan Apochromat NA 1.4, WD 0.19), with 1μm z-steps at 3-minute intervals.

For live imaging of 3D spheroids, spheroids were established as described above. Prior to live imaging, SiR-DNA (Tebu-Bio) was added to spheroids at 1:5000 dilution for 2 hours. Media was then replaced with fresh Assay Medium containing ethanol or 4-OHT. Time-lapse fluorescence imaging was performed on the Nikon W1 spinning disc using 20x/0.75NA Air objective with images taken every 5 minutes for at least 16 hours at 37°C with 5% CO_2_.

### Cell fixation and immunostaining

For immunofluorescence imaging, cells were plated 24 hours prior to instituting the experimental conditions and subsequent fixation on fibronectin-coated dishes (Labtek). Cells were fixed using 4% paraformaldehyde (PFA) and incubated at room temperature for 20 minutes. Cells were permeabilised using 0.2% Triton X in PBS for 5 minutes. 5% bovine serum albumin (BSA)/PBS was used to block non-specific binding for 30 minutes at room temperature. Cells were incubated with primary antibodies (α-tubulin-FITC Sigma 1:400) or fluorescent conjugated small molecules (DAPI 1:1000 Invitrogen, Phalloidin-TRITC 1:2000 Sigma) in 1% BSA/PBS for 1 hour at room temperature. Spheroid fixation and immunostaining followed the same protocol. Fixed samples were imaged on a Leica TCS SPE 2 microscope using 63x objective (ACS APO 63x oil NA1.3 DIC=E, coverslip correction 0.17). AiryScan confocal imaging was carried out using a Zeiss LSM 880 AiryScan Confocal and a Zeiss LSM980 Airyscan 2 Confocal.

### Cell culture on hydrogels of different stiffness

Polyacrylamide (PAA) hydrogels of different stiffness were prepared as previously described.^33^ Glass-bottom dishes (Ibidi) were treated with 1M NaOH before being cleaned with ethanol and incubated with Bind-Silane (Abbexa) for one hour. PAA gel mixes were prepared by mixing 40% acrylamide, 2% bis-acrylamide with 0.5% of 10% ammonium persulfate and 0.05% of N,N,N,N’-te-tramethylethylenediamine in PBS. The following acrylamide/bis-acrylamide ratios were used for different stiffness gels: ~12 kPa: 18.8% acrylamide/8 % bis, ~6 kPa: 18.7% acrylamide/3 % bis, ~2 kPa: 13.7% acrylamide/3.5 % bis and ~0.7 kPa: 10% acryl-amide/1.5 % bis. 22 μL of PAA solution was pipetted onto the treated dish, covered with an 18mm coverslip and left to polymerise for 1 hour. After polymerisation, PBS was added to gels and coverslips removed. PAA gels were then functionalised by Sulfo-SANPAH (1 mg/ml; sulfosuccinimidyl 6-(4‘-azido-2’-nitrophenylamino) hexanoate) treatment for 5 min under an ultraviolet lamp at 365-nm. Gels were then washed with PBS before overnight incubation with 0.5mg/ml collagen type I at 37°C. Gels were pre-incu-bated with media before addition of cells in a 50 μL droplet.

## Quantification And Statistical Analysis

### Image analysis

Images were processed and analysed using Fiji/ImageJ version 1.0.^57^ Confocal fluorescent images are displayed as single plane or maximum intensity projections, as indicated in the figure legends. For cell shape analyses, outlines were manually segmented from bright-field or confocal images using the polygon selections tool in Fiji. Spindle angle analyses were obtained as the angle between the x-axis and a line drawn between the two spindle poles segmented from maximum projections of confocal images. Retraction fibre quantification was performed using the multi-point tool in Fiji. Retraction fibres were counted for isolated cells by their most peripheral point. Broken retraction fibres were defined as the peripheral point not being in continuity with the cell body. For spheroid shape analyses, outlines were manually segmented from confocal images using the polygon selections tool in Fiji. Image overlays were created using the Matplotlib library in Python. For division orientation analysis within 3D spheroids a line from the centre of the spheroid was drawn to the spheroid periphery intersecting the dividing cell. A line parallel to the anaphase chromosomes was drawn and the difference between these two lines was calculated to determine the division angle relative to the spheroid edge. Images of overlaying cell outlines were produced using CellProfiler version 3.1.8 software^58^ and overlay pipelines. 3D rendering of AiryScan confocal images was carried out using Arivis Vision4D (Zeiss).

For calculations of the Jaccard similarity and dissimilarity indices, the standard equations^28^ were implemented using custom-made scripts in Python using the NumPy,^59^ scikit-image^60^ and SciPy libraries.^61^ To obtain the Jaccard index with shape contribution only, the areas of the cell outlines were made equal by scaling (i.e. a uniform change in size without altering shape) one of the images. The contribution of cell displacement was then removed by translating (i.e. moving the image without any change in shape or size) one image so that its centroid matched the centroid of the other image. Finally, to adjust for the contribution of changes in cell orientation during mitosis, the shape was rotated about the centroid until a maximal value of Jaccard index was found, at the angle at which they aligned. Cell orientation was calculated from the central moments of the binary mask segmentation of the cell.

### Statistical analysis

Graphs were produced in Graphpad Prism (version 9.1.2). Bar charts and scatter plots show mean with error bars showing standard deviation. Stacked bars of summary data indicate mean percentages totalling 100% with error bars showing standard deviation. Where indicated, data was pooled from independent experiments, where N = number of independent experiments. The number of cells (n) analysed in each condition is indicated in parentheses on plots. Statistical tests were carried out in Graphpad Prism. All data sets were tested for normality using the D’Agostino-Pearson test for normality. For normal data sets, student T-tests were used to test whether differences were statistically significant. All non-normal data sets were analysed using Mann-Whitney two-tailed test. *p<0.01 **p<0.001 ***p<0.0001 ****p<0.00001.

## Supplementary Material

Figs S1-S5

Video S1

Video S2

Video S3

Video S4

## Highlights

Oncogenic Ras alters cell shape dynamics during post-mitotic respreading
High contractility in Ras-activated cells breaks mitotic substrate contacts
Loss of substrate cues lead to defects in mitotic spindle orientation
Ras induces out-of-plane divisions in epithelial monolayers and spheroids

## In brief

Cell division is dysregulated in cancer. Ganguli et al. show that activation of an oncogene, Ras, directly alters cell-substrate adhesion and cell shape during progression through mitosis. These changes are accompanied by alterations in mitotic spindle orientation, which lead to out-of-plane divisions and loss of epithelial tissue architecture.
