# Supplementary material for: Oncogenic Ras deregulates cell-substrate interactions during mitotic rounding and respreading to alter cell division orientation
Source: Curr Biol. Author manuscript; Available in PMC 2023 Aug 4. (PMC7614879; doi:10.1016/j.cub.2023.05.061)
Supplement: Figs S1-S5 [file EMS182025-supplement-Figs_S1_S5.pdf]

Current Biology, Volume *33*

Supplemental Information

Oncogenic Ras deregulates cell-substrate interactions during mitotic rounding and respreading to alter cell division orientation

Sushila Ganguli, Tom Wyatt, Agata Nyga, Rachel H. Lawson, Tim Meyer, Buzz Baum, and Helen K. Matthews

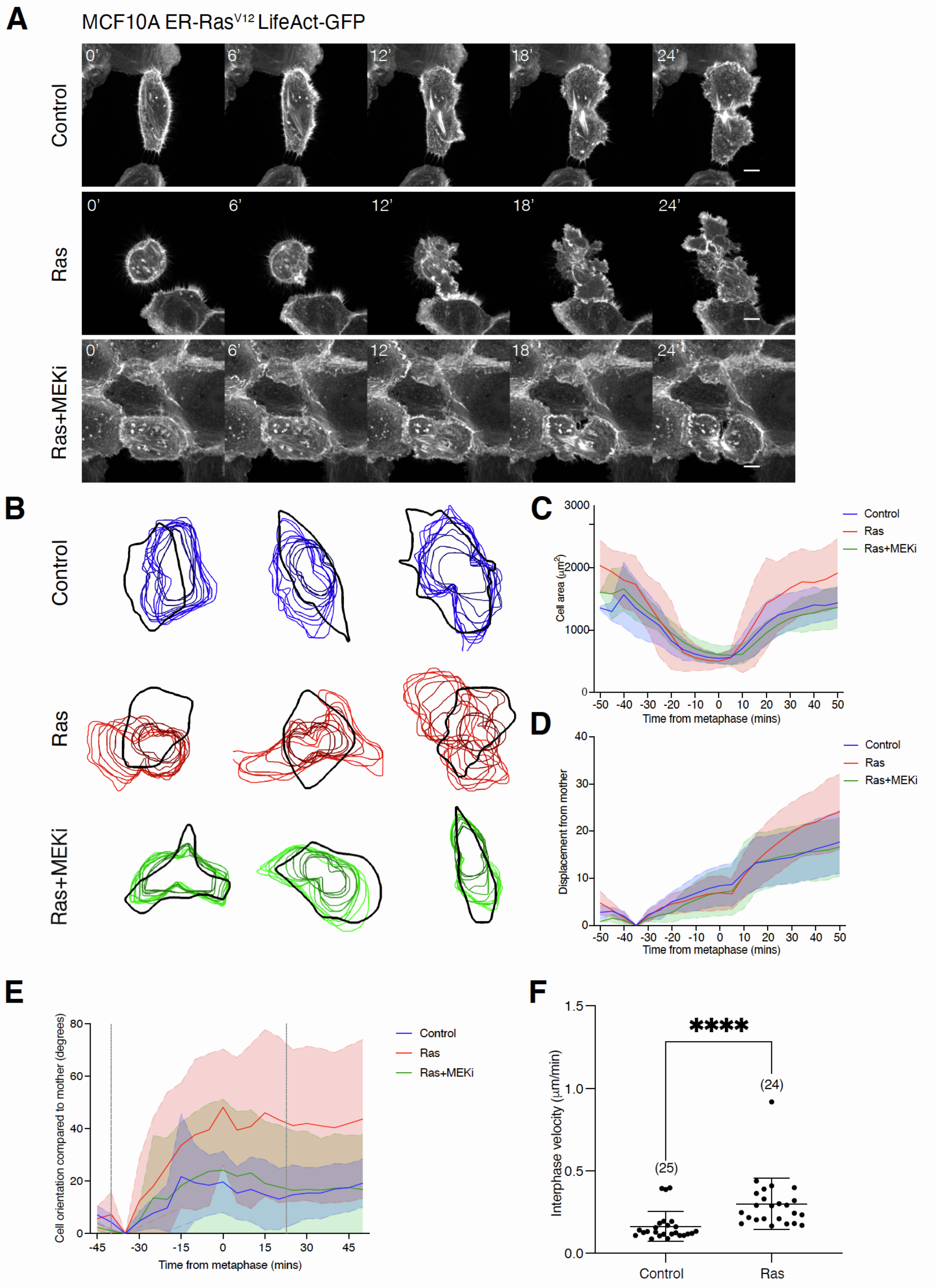

A
MCF10A ER-Ras^{V12} LifeAct-GFP
Control
Ras
Ras+MEKi
0'
6'
12'
18'
24'
B
Control
Ras
Ras+MEKi
C
Cell area (µm²)
Time from metaphase (mins)
Control
Ras
Ras+MEKi
D
Displacement from mother
Time from metaphase (mins)
Control
Ras
Ras+MEKi
E
Cell orientation compared to mother (degrees)
Time from metaphase (mins)
Control
Ras
Ras+MEKi
F
Interphase velocity (µm/min)
****
(25)
(24)
Control
Ras

Figure S1. Analysis of post-mitotic respreading in MCF10A-LifeActGFP-ER-Ras^{V12} cells (related to figure 1)

(A) Time-lapse images of representative ER-Ras^{V12} cells labelled with LifeAct-GFP exiting mitosis. Cells were treated with a 5-hour treatment of ethanol, 4-OHT or 4-OHT + 10 µM selumetinib before imaging every 3 minutes. Images are presented as maximum projections. Time is aligned so t=0 represents the frame 3 minutes before anaphase. Scale bars represent 10µm.

(B) Cell outlines as cells respread post-mitosis with respect to the mother cell shape. MCF10A-LifeAct-GFP-ER-Ras^{V12} cells were treated with ethanol, 4-OHT or 4-OHT plus 10µM selumetinib for 5-15 hours prior to time-lapse bright-field and fluorescence imaging every 5 minutes. Representative dividing cells were manually segmented at interphase (black), defined using the bright-field channel as 15 minutes before nuclear envelope breakdown (NEB). Post-mitotic respreading cell outlines were segmented at 5-minute intervals for 10 consecutive frames from the final frame of metaphase following ethanol (blue), 4-OHT (red) or 4-OHT plus MEKi (green). Image overlays were produced using CellProfiler software as described in Methods.

(C) Graph to show the cell area of ER-Ras^{V12} cells treated with the conditions described in (B) as they progress through mitosis at consecutive time-points. n= 10 cells.

(D) Graph to show centroid displacement of cells with respect to the mother of cells treated with the conditions described in (B) as they progress through mitosis. n= 10 cells.

(E) Graph to show the cell shape orientation of ER-Ras^{V12} cells treated with the conditions described in (B) as they progress through mitosis at consecutive time-points. n= 10 cells.

(F) Quantification of interphase cell velocity. Cell tracking measurements were taken for cells following treatment with ethanol (control) or 4-OHT every 10 minutes for 4 hours. Mean cell velocity is plotted. N=3 experiments.

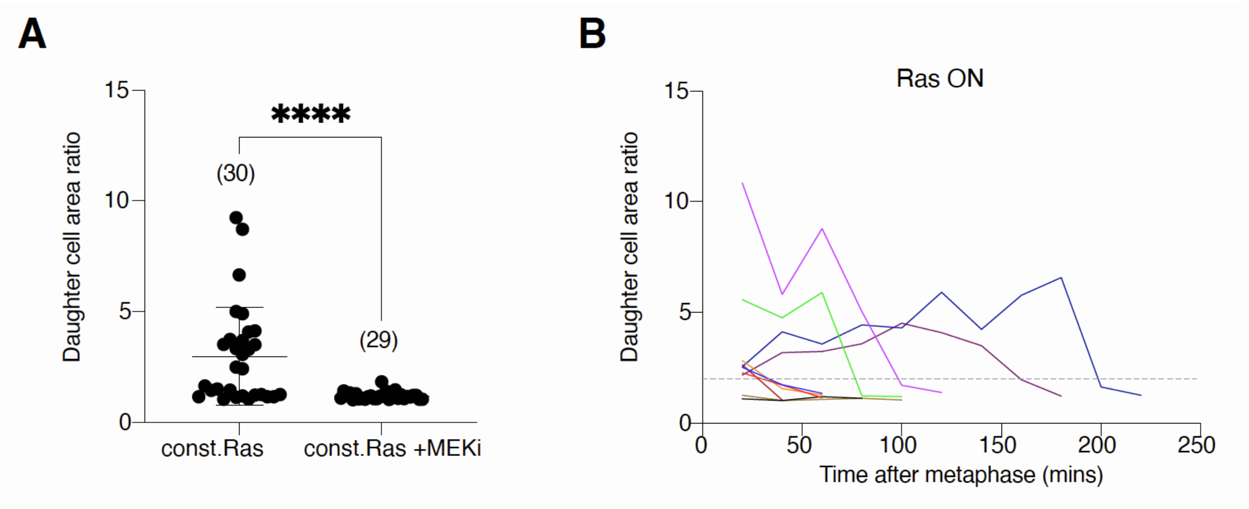

Figure S2. Analysis of daughter cell asymmetry in MCF10A cells with stable and inducible Ras^{V12} expression (related to figure 2)

(A) Plot of daughter cell area ratio of MCF10A cells constitutively expressing Ras^{V12} following DMSO (control) or selumetinib (MEKi) treatment. N=3 experiments.

(B) Graph to show daughter cell area ratio over time as cells exit mitosis for 10 individual MCF10A-ER-Ras^{V12} cells following 4-OHT treatment. Dotted line indicates daughter cell area ratio of 2.

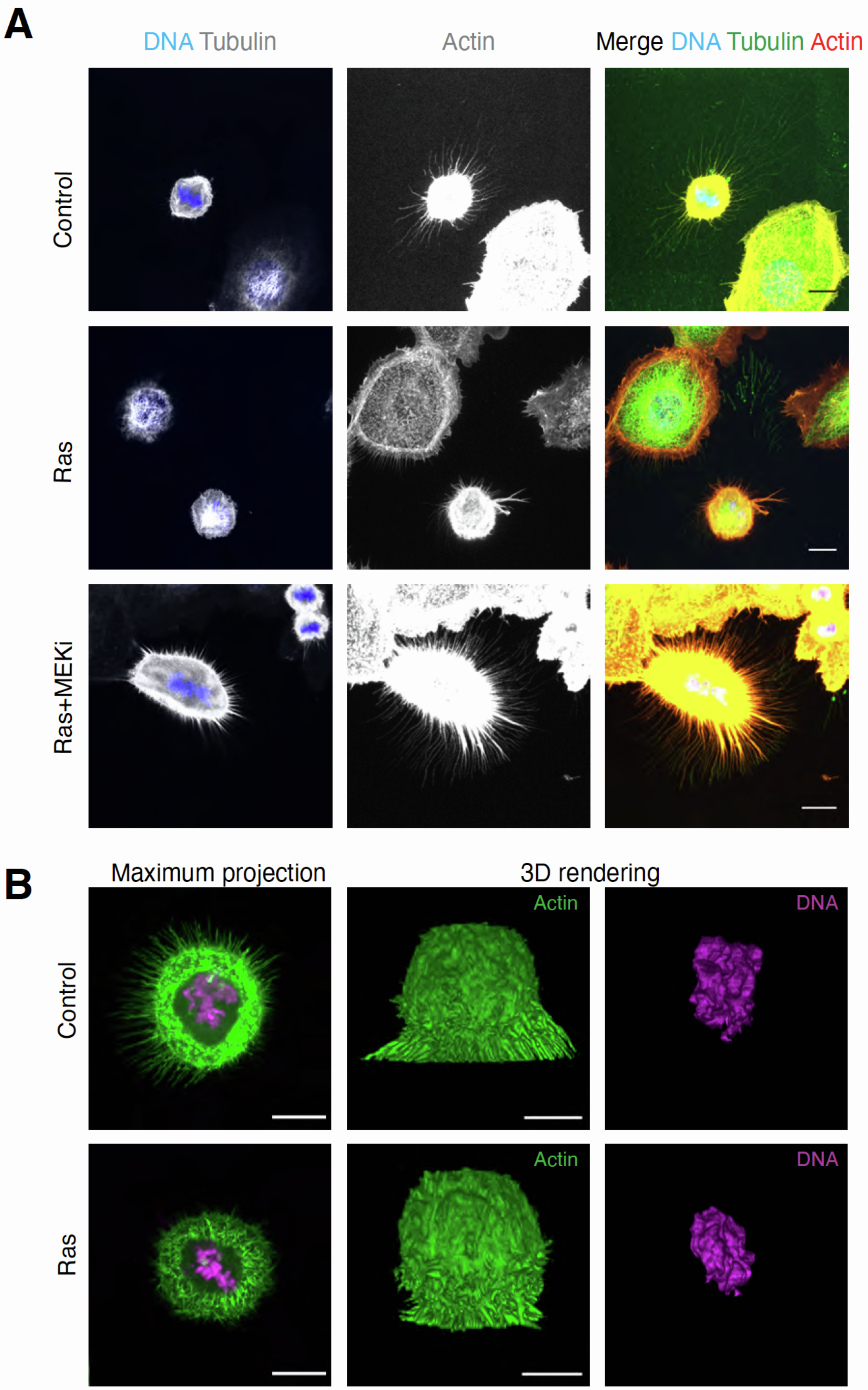
A
DNA Tubulin
Actin
Merge DNA Tubulin Actin
Control
Ras
Ras+MEKi
B
Maximum projection
3D rendering
Actin
DNA
Control
Actin
DNA
Ras

Figure S3. Immunofluorescence imaging of retraction fibres in MCF10A-ER-Ras^{V12} cells (related to figure 4)

(A) Immunofluorescence images of MCF10A-ER-Ras^{V12} cells in metaphase following a 5-hour treatment of ethanol, 4-OHT or 4-OHT+ 10 µM selumetinib (MEK inhibitor). Cells are stained with DAPI, monomeric α-tubulin and phalloidin-TRITC. DAPI and tubulin images are displayed as a single z-slice, actin and merge images are displayed as maximum projections. Scale bars represent 10µm.

(B) AiryScan confocal images of MCF10A-ER-Ras^{V12} LifeAct-GFP cells in metaphase following a 7-hour treatment of ethanol (Control) or 4-OHT (Ras). DNA is visualised using SiR DNA (Spirochrome). Images show maximum projection of the lower half of the cell (substrate to centre) and 3D surface rendering of actin and DNA channels. Scale bars represent 10µm.

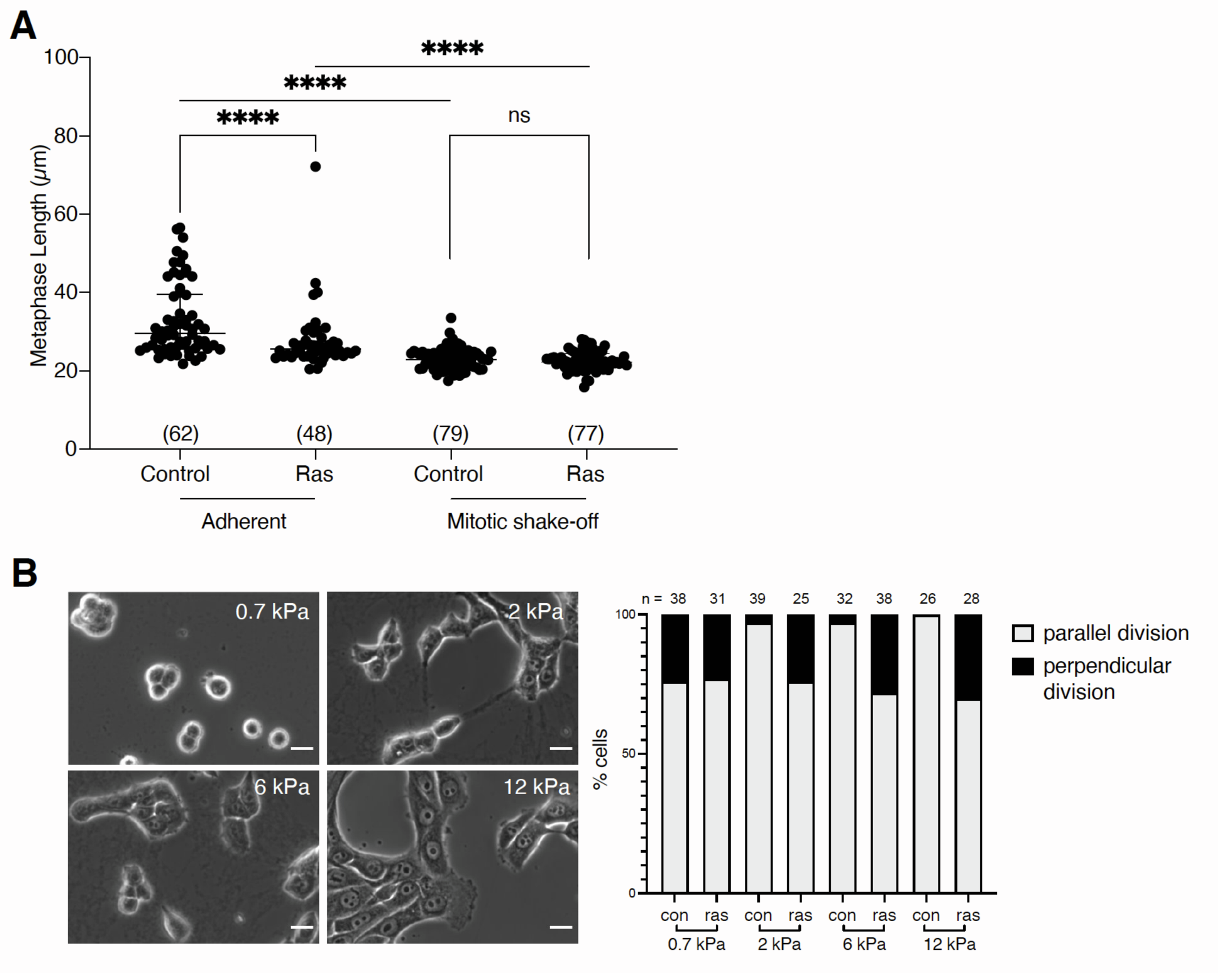

Figure S4. Analysis of cell division following mitotic shake-off and on different stiffness hydrogels (related to figure 4)

(A) Plot of individual metaphase cell length (Feret) measurements (taken as the frame 5 minutes before anaphase elongation) from the mitotic shake off experiment described in Fig. 4e. N= 3 experiments.

(B) Images of control cells plated on polyacrylamide hydrogels of different stiffnesses. Note that cells spread on all stiffnesses except 0.7 kPa. Graph shows quantification of the percentage of cells dividing parallel and perpendicular to the substrate following 5-15 hours treatment with ethanol (con) or 4-OHT (Ras). Division orientation was followed using tubulin channel from timelapse movies of MCF10A-ER-Ras^{V12}-tubulin-GFP cells. N = 2 experiments. Scale bars are 20µm.

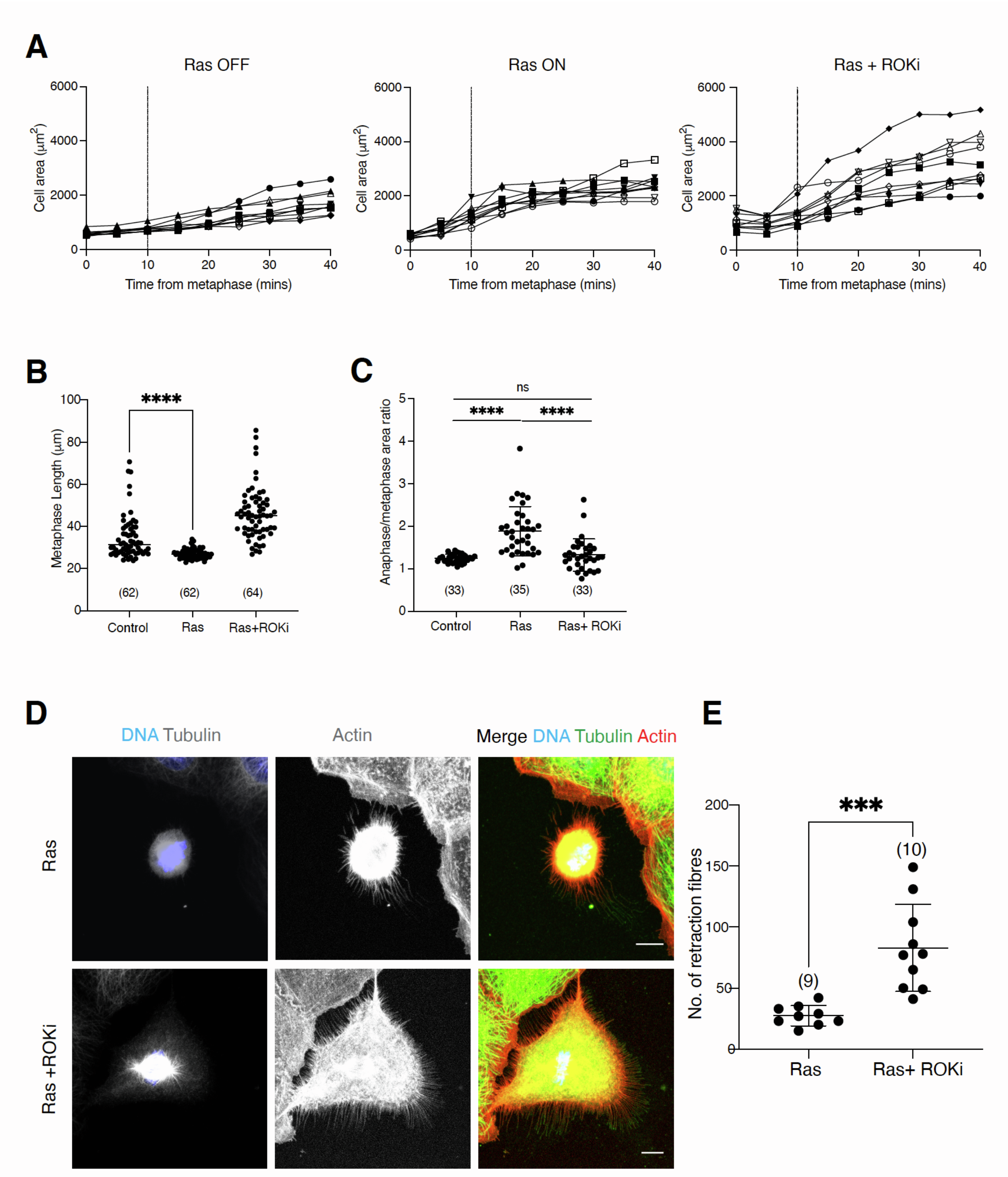

Figure S5. Analysis of cell shape and retraction fibres following ROCK inhibition (related to figure 5)

(A) Quantification of cell area for 10 ER-Ras^{V12} cells exiting mitosis following ethanol, 4-OHT or 4-OHT plus 25µM Y27632 (ROCK inhibitor). Measurements were taken from phase-contrast time-lapse microscopy images of cells every 5 minutes following 5–15 h of treatment. Dotted lines indicate 10 minutes after the onset of anaphase from which statistical analysis is calculated in (C).

(B) Plot of individual metaphase cell length (Feret) measurements from the ROCK inhibitor experiment described in Fig. 5c. N= 3 experiments.

(C) Plot of the ratio of cell area (10 minutes following anaphase onset/ metaphase) for ER-Ras^{V12} cells following ethanol, 4-OHT or 4-OHT plus 25µM Y27632 (ROCK inhibitor) treatment. N= 3 experiments.

(D) Immunofluorescence images of MCF10A-ER-Ras^{V12} cells in metaphase following a 5-hour treatment of 4-OHT or 4-OHT+ 25µM Y27632 (ROCK inhibitor). Cells are stained with DAPI, monomeric α-tubulin and phalloidin-TRITC. DAPI and tubulin images are displayed as a single z-slice, actin and merge images are displayed as maximum projections. Scale bars represent 10µm.

(E) Quantification of the total number of retraction fibres in single cells from the conditions described in (D).
